# Alginate/Chitosan Particle-Based Drug Delivery Systems for Pulmonary Applications

**DOI:** 10.3390/pharmaceutics11080379

**Published:** 2019-08-02

**Authors:** Marcus Hill, Matthew Twigg, Emer A. Sheridan, John G. Hardy, J. Stuart Elborn, Clifford C. Taggart, Christopher J. Scott, Marie E. Migaud

**Affiliations:** 1 School of Pharmacy, Queen’s University Belfast Queen’s University Belfast, Belfast BT7 1NN, UK; 2 Airway Innate Immunity Group (AiiR), Wellcome Wolfson Institute of Experimental Medicine, School of Medicine, Dentistry and Biomedical Sciences, Queen’s University Belfast, 97 Lisburn Road, Belfast BT9 7BL, Northern Ireland, UK; 3 Lancashire Teaching Hospitals NHS Foundation Trust, Royal Preston Hospital, Sharoe Green Lane PR2 9HT, UK; 4 Department of Chemistry, Lancaster University, Lancaster, Lancashire LA1 4YB, UK; 5 Materials Science Institute, Lancaster University, Lancaster, Lancashire LA1 4YB, UK; 6 School of Medicine, Dentistry and Biomedical Sciences, Queen’s University Belfast, 97 Lisburn Road, Belfast BT9 7BL, Northern Ireland, UK; 7 Centre for Cancer Research and Cell Biology, School of Medicine, Dentistry and Biomedical Sciences, Queen’s University Belfast, 97 Lisburn Road, Belfast BT9 7BL, Northern Ireland, UK; 8 USA Mitchell Cancer Institute, University of South Alabama, Mobile, AL 36604, USA

**Keywords:** biomedical applications, drug delivery systems, particles, antimicrobial

## Abstract

Cystic fibrosis (CF) is a complex, potentially life-threatening disease that is most effectively treated through the administration of antibiotics (e.g., colistimethate sodium). Chronic infection with *Pseudomonas aeruginosa* is one of the most significant events in the pathogenesis of cystic fibrosis, and tobramycin is the treatment of choice for those patients with chronic *P. aeruginosa* infection who are deteriorating despite regular administration of colistimethate sodium. Effective treatment can be challenging due to the accumulation of thickened mucus in the pulmonary environment, and here we describe the results of our investigation into the development of alginate/chitosan particles prepared via precipitation for such environments. Tobramycin loading and release from the alginate/chitosan particles was investigated, with evidence of both uptake and release of sufficient tobramycin to inhibit *P. aeruginosa* in vitro. Functionalisation of the alginate/chitosan particles with secretory leukocyte protease inhibitor (SLPI) was shown to help inhibit the inflammatory response associated with lung infections (via inhibition of neutrophil elastase activity) and enhance their interaction with cystic fibrosis mucus (assayed via reduction of the depth of particle penetration into the mucus) in vitro, which have prospects to enhance their efficacy in vivo.

## 1. Introduction

**Figure 1:**
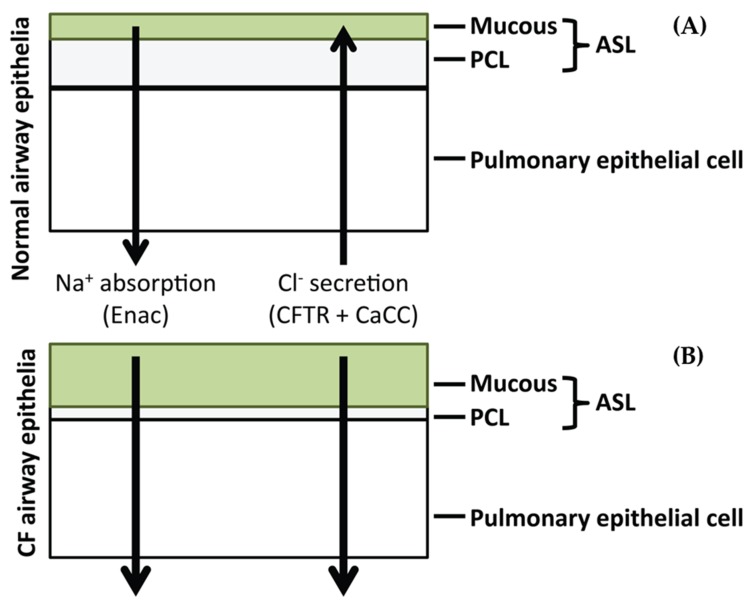
Events linking altered lung airway surface layer (ASL) volumes to decreased mucociliary clearance. (**A**) Normal airway surfaces contain a small mucus layer which facilitates entrapment of inhaled particles and pathogens. ASL autoregulation leads to the maintenance of the pericilliary liquid layer (PCL) which allows movement and clearance of inhaled particles and pathogens. (**B**) Hyperabsorption of Na^+^ and ineffective Cl^−^ secretion in the cystic fibrosis (CF) airway cause depletion of ASL, collapse of the cilia in the PCL and adherence of concentrated mucus in the airways.

Cystic fibrosis (CF) is a complex, potentially life-threatening disease which is manifested through mutations in the cystic fibrosis transmembrane conductance regulator (CFTR) [1]. The protein product of this gene functions as a cyclic adenosine monophosphate (cAMP)-dependent transmembrane chloride (Cl^−^) channel. It is responsible for the transport of ions across the apical membrane of exocrine epithelial cells [2]. The protein shows widespread expression across the epithelial lining of most exocrine glands, though it is mainly expressed in cells of the small intestine, airways, vas deferens and ducts of the pancreas [3,4,5,6]. The mutations present in the CFTR gene have been shown to lead to the subsequent loss of Cl^−^ transport in these cell types. The pulmonary environment has been shown to be one of the most largely effected by the loss of Cl^−^ channel activity [7] concomitant with a decrease in volume of the airway surface liquid (ASL) causing the accumulation of thickened mucus in the pulmonary environment (Figure 1) [8,9]. This is believed to increase the susceptibility of the lung to infection by organisms including *Pseudomonas aeruginosa*, *Haemophilus influenza* and *Staphylococcus aureus* [10]. Early infecting strains such as *S. aureus* are usually cleared by antibiotic therapy, though they are believed to facilitate chronic colonisation by *P. aeruginosa* [11]. Chronic colonisation with *P. aeruginosa* coincides with increasing antibiotic resistance and the emergence of *P. aeruginosa* as the dominant infecting organism over time [12].

Chronic infection with *P. aeruginosa* is one of the most significant events in the pathogenesis of cystic fibrosis [13]. Tobramycin is the treatment of choice for those patients with chronic *P. aeruginosa* infection who are deteriorating despite regular colistimethate sodium [14,15]. It is one of the most effective treatments for pulmonary exacerbations and is generally delivered through nebulisation at a dose of 300 mg twice daily. The pharmacokinetic parameters are similar to other aminoglycoside drugs resulting in rapid renal excretion [16]. As with other aminoglycosides, tobramycin shows no activity against gram positive organisms such as *S. aureus*, therefore early CF antibacterial therapies often focus on administration of anti-staphylococcal prophylactic antibiotics [17].

Clinical treatment with tobramycin involves twice daily administration of high doses of the drug due to its high absorption and short plasma half-life [18]. One of the main problems with CF therapy is non-compliance. Non-compliance with antibiotic regimens can lead to antibiotic failure [19], therefore altering the dosing schedule of tobramycin from twice daily to once daily may in part address compliance issues. Another concern with prolonged tobramycin treatment is the potential toxicity experienced due to the ototoxic and nephrotoxic property of the drug [20,21]. Nephrotoxicity is often associated with parenteral aminoglycoside therapy, but there is limited evidence of nephrotoxicity and ototoxicity in clinical trials with nebulised tobramycin [22]. With longer durations of therapy there is an increased potential risk of nephrotoxicity. Therefore, the development of delivery systems for tobramycin which could reduce the dosing frequency of the drug while also reducing systemic toxicity upon prolonged exposure may be of clinical benefit.

The CF lung provides a further barrier to efficient drug delivery due to the presence of a thick mucus layer on the epithelial lining [23]. This is due to the altered salt transport system within the CF lung, which results in increased dehydration and mucus viscosity with delayed mucus clearance [24]. Furthermore, *P. aeruginosa* forms a thick alginate-based biofilm which provides increased resistance to antibacterial therapy [11].

A further complication arises from the enhanced pro-inflammatory response to infection which causes increased infiltration of neutrophils to the site of bacterial colonisation [25]. Neutrophils promote the pro-inflammatory response to infection through the release of various pro-inflammatory proteases such as neutrophil elastase (NE) which can overload the endogenous anti-protease host defenses, leading to tissue damage and loss of respiratory function [12]. There is therefore interest in the development of drug delivery systems which can enhance the antibacterial activity of the delivered payload through increased accumulation at the site of infection while reducing the aberrant inflammatory response to infection.

The secretory leukocyte protease inhibitor (SLPI) is an 11.7 kDa protein which is naturally expressed as part of the innate immune response in humans [26]. SLPI is naturally expressed in a number of bodily secretions including nasal, pulmonary, salivary and seminal secretions [27,28,29,30]. SLPI has been shown to display potent anti-inflammatory activity through inhibition of a number of endogenous serine proteases including NE [31]. Due to the high association rate constant for NE it is believed SLPI acts as the primary inhibitor of NE in vivo [12]. Studies have shown therapeutic administration of recombinant SLPI (r-SLPI) to CF patients can reduce the inflammatory response by lowering active levels of NE while also reducing NE mediated interleukin 8 (IL-8) production, which is involved in the recruitment of pro-inflammatory mediators to the site of disease [32,33]. SLPI has also been shown to reduce inflammation through inhibition of nuclear factor kappa-light-chain-enhancer of activated B cells (NF-ĸB) signaling [34].

Here we describe the results of our investigation on the development of alginate/chitosan particles for pulmonary applications. In the first paradigm, tobramycin loading and release was investigated, and in the second paradigm the particles were functionalised with SLPI to help inhibit the inflammatory response associated with infection (and potentially passively target the particles through binding with anionic mucins within mucus to provide enhanced targeting to the site of disease due to its cationic nature).

## 2. Materials and Methods

### 2.1. Materials

Unless otherwise noted, all chemicals (including Calbiochem neutrophil elastase substrate (methoxysuccinyl-l-alanyl-alanyl-prolyl-l-valyl-4-nitroanilide)) were obtained from Sigma Aldrich, Gillingham, UK. Boric acid, calcium chloride hexahydrate, ethanol and the Pierce bicinchoninic acid (BCA) protein assay kit were purchased from Fisher Scientific UK, Loughborough, UK. Tryptone, sodium chloride (bacteriological grade) and yeast extract were purchased from Oxoid Ltd., Basingstoke, England. Recombinant human neutrophil elastase was purchased from the Elastin Products Company (EPC) Owensville, MO, USA. Recombinant human secretory leukocyte protease inhibitor (SLPI) was purchased from Amgen, Cambridge, UK. Biotinylated anti-human SLPI antibody was purchased from R&D Systems, Abingdon, UK. Sputum from CF patients was obtained anonymously from the adult CF centre at Belfast City Hospital. Sputum samples were in excess to requirements for diagnostic purposes. Permission to use sputum samples (which would have been disposed of) for validation purposes was given by the director of R&D in Belfast Health and Social Care Trust.

### 2.2. Preparation of Tobramycin Loaded Alginate/Chitosan Particles

Several formulations of tobramycin were tested for their ability to formulate tobramycin loaded particles with no aggregation (Table 1). CaCl_2_ and chitosan were utilised as cationic crosslinkers. Tobramycin was included in all formulations. Briefly, aqueous solutions of tobramycin (1.5 mg in 3 mL) were added to various amounts of sodium alginate (pH 5.4) while stirring (500 rpm) at room temperature. 1 mL of chitosan (1% *v/v* glacial acetic acid, pH 5.1) at various concentrations was added dropwise (over ca. 5 min) from a syringe fitted with a 25G needle while stirring (500 rpm). The particle suspension was collected by centrifugation for 30 min at 20,000 g, after which the pellet of particles was resuspended in PBS and collected by centrifugation for 30 min at 20,000 g, this resuspension/centrifugation process was repeated twice more.

### 2.3. Particle Characterisation

The size distribution (polydispersity index; PDI) and zeta potential of the particles was determined by dynamic light scattering (DLS) using a Malvern Zetasizer (Nano ZS; Malvern instruments, Malvern, UK). For particle analysis, each sample was read in triplicate (10 runs each). The average of three separate samples was taken and the data presented as the mean ± standard deviation. Transmission electron microscopy (TEM) was performed using a JEOL JEM1400 (JEOL, Inc. Peabody, MA, USA) transmission electron microscope at an accelerating voltage of 80 kV. Particles (0.6 mg/mL) were loaded on a copper grid (Formvar/Carbon 200 µm mesh, Agar Scientific Ltd., Stansted, UK), the moisture wicked off and allowed to dry. 2 µL of uranyl acetate (2% *w*/*v*) was added to provide contrast between the particles and copper grid.

### 2.4. Conjugation of SLPI to Chitosan

Chitosan 0.5 mL (2 mg/mL, in 1% *v/v* glacial acetic acid) was diluted 1:1 in PBS buffer (25 mM, pH 7.4) and 10 µL of SLPI was added (25 mg/mL). The pH of the solution was adjusted to 5 and the conjugation was initiated by the addition of 1-ethyl-3-(dimethylaminopropyl carbodiimide) (EDC) (2.5 mg/mL) and left stirring for 6 h at room temperature. The SLPI/chitosan conjugate was used instantly for particle formation with alginate and excess EDC was removed by washing of the particles through centrifugation/resuspension cycles (assaying for EDC via thin layer chromatography, TLC).

### 2.5. Quantification of SLPI Conjugation

Particles were formulated as described previously and centrifuged at 20,000 g. The supernatant was collected. Blank particles (not conjugated to SLPI) were also included as a control. The levels of SLPI conjugation achieved were measured with the BCA assay kit. 25 µL of control and conjugate particles (10 mg/mL) were added to 175 µL of BCA reagent A (sodium carbonate, sodium bicarbonate, bicinchoninic acid and sodium tartrate in 0.1 M sodium hydroxide) and B (4% W/W cupric sulphate in water) in a 96 well plate. The plate was then incubated for 60 min at 37 °C. The absorbance of the resultant solution was read at 570 nm and compared to a calibration curve of SLPI.

### 2.6. Analytical Methodology for Detection of Tobramycin Sulphate

Reagent A, consisting of 80 mg of ortho-phthaldialdehyde in 1 mL of 95% ethanol, and reagent B, containing 200 µL of boric acid (pH 9.7, 0.4 M), 400 µL β-mercaptoethanol and 200 µL of diethyl ether were mixed. Serial dilutions of tobramycin were prepared in boric acid (pH 9.7, 0.4 M). 100 µL of each tobramycin standard was added to 100 µL of the reagent mixture. The plate was read by fluorescence at λ_ex_/λ_em_ 360/460 nm, respectively.

### 2.7. Quantification of Tobramycin Release

Drug release was quantified by incubating the particles in dialysis membranes with a 10,000 Da MWCO (Thermo Fisher Scientific, Heysham, UK) at 37 °C with agitation. The release of the tobramycin was quantified by incubating 3 mg of particles in 1 mL of PBS in the donor compartment with 5 mL of PBS in the receiver compartment. At each time point the PBS was collected and replaced with fresh PBS release medium. Quantification of the tobramycin release was performed by diluting the release medium 1:1 with boric acid (0.4 M pH 9.7) prior to derivatisation with ortho-phthaldialdehyde (80 mg) in a solution containing 1 mL of 95% ethanol, 200 µL boric acid (0.4 M pH 9.7), 400 µL of β-mercaptoethanol and 200 µL diethyl ether. The fluorescent derivative was monitored by fluorescence at λ_ex_/λ_em_ 360/460 nm, respectively using a BMG-LABTECH Fluorstar Optima fluorescent plate reader (BMG-LABTECH, Aylesbury, UK).

### 2.8. Minimum Inhibitory Concentration Effect of Tobramycin on P. aeruginosa

Broth micro-dilution tests were performed according to National Committee for Clinical Laboratory Standards (NCCLS) guidelines. Serial two-fold dilutions of tobramycin (from stock solution which had been sterile filtered with 0.22 µm filter) in 100 µL of Luria Bertani (LB) broth were performed on a 96 well plate in the range 0–25 µg/mL for the drug loaded particles, free tobramycin and the blank particles as negative control. The actively growing cultures were diluted to an optical density reading of 0.3 (A550) to give a starting inoculum of 2 × 10^5^ CFU/mL. 100 µL of the starting inoculum (2 × 10^5^ CFU/mL) was added to each well of the plate and incubated aerobically at 37 °C for 24 h. Positive and negative controls were included in each assay.

### 2.9. Inhibition of Neutrophil Elastase by SLPI Functionalised Alginate/Chitosan Particles

Anti-neutrophil elastase activity was monitored by incubation with the human neutrophil elastase (HNE) specific substrate methoxysuccinyl-Ala-Ala-Pro-Val-P-nitroanilide (Sigma, Aldrich, UK). Inhibitions of HNE (Elastin products, Owensville, MO, USA) were measured in the presence and absence of both blank and SLPI conjugated particles. The assay was carried out by incubating 10 µL (10 mg/mL particles) of blank and SLPI conjugated particles containing 100 µg/mL SLPI with 4 µL of HNE (100 µg/mL). HNE activity was measured by the cleavage of chromogenic methoxysuccinyl-Ala-Ala-Pro-Val-P-nitroanilide substrate by adding 50 µL of 0.2 mM substrate in 0.1 M HEPES buffer containing 0.5 M NaCl. Assays were conducted at 37 °C and the formation of the fluorescent product (*p*-nitroaniline) was measured continuously at 405 nm on a BMG-Labtech Fluorstar Optima fluorescent plate reader.

### 2.10. Preparation of Rhodamine 6G Loaded Alginate/Chitosan Particles

3 mL of a tobramycin stock solution in water (0.5 mg/mL) was added to 3 mL of sodium alginate (3 mg/mL pH 5.4). 250 µL of rhodamine 6G (2 mg/mL) was then added to the tobramycin alginate mixture. 1 mL of chitosan (1% *v/v* glacial acetic acid pH 5.1) was added dropwise from a syringe fitted with a 25G needle while stirring (500 rpm). The particle suspension was collected by centrifugation for 30 min at 20,000 g, after which the pellet of particles was resuspended in PBS and collected by centrifugation for 30 min at 20,000 g, this resuspension/centrifugation process was repeated twice more.

### 2.11. Penetration of SLPI Functionalised Particles in CF Mucus

The penetration of rhodamine loaded particles with and without SLPI functionalisation was studied in CF mucus. A 500 µL layer of 10% gelatin (Porcine type A Sigma- Aldrich) was added to a 24 well microplate and allowed to harden. 500 µL of CF sputum or PBS control was added and allowed to settle. 200 µL of SLPI functionalised particles (10 mg/mL) were added to the sputum and PBS control wells with SLPI at a concentration of 100 µg/mL. Non-conjugated rhodamine loaded particles were diluted to give an equivalent concentration of rhodamine. Particle penetration was measured over 24 h after which the gelatin layers were washed (PBS × 6) and the gelatin was melted and fluorescence measured at 480/520 nm. Penetration in CF mucus was measured by comparison to the PBS control which was measured as 100% penetration. Fluorescent values were analysed in reference to rhodamine 6G standards in the range (0–1000 ng/mL).

### 2.12. Statistical Analysis

Experiments were repeated in triplicate and reported as mean ± standard deviation (S.D.). Results were analysed with GraphPad Prism, version 8.02, GraphPad Software (San Diego, CA, USA). T-test analysis was performed as appropriate. Statistical significance critical values were defined as ** *p* < 0.01, *** *p* < 0.0001.

## 3. Results and Discussion

We have an interest in the development of particles designed for therapeutic application in diseases such as cystic fibrosis (CF) [35].

### 3.1. Particle Preparation and Tobramycin Loading and Release

Various particle-based drug delivery systems have been developed to facilitate the effective delivery of drugs to the pulmonary system [36]. Here we report the development of tobramycin-loaded particles composed of alginate and chitosan. The results of varying the ratio of the components (alginate:chitosan:tobramycin:CaCl_2_) on particle formation are reported in Table 1.

During the optimisation studies we observed that a higher concentration of cations in the formulation resulted in the aggregation of the particles (Table 1). The optimal formulation of alginate:chitosan:tobramycin 9:1:1.5 (*w*/*w*/*w*), resulted in the formation of particles with a fairly narrow size distribution and high loading of tobramycin (Table 2, Figures S1 and S2) and was therefore used for further studies. The particle size distribution assessed by DLS measurements (Table 2, Figure S1) were somewhat larger than those observed by TEM (Figure S2), however, the DLS data was judged to be more representative of the whole population of particles as the particles are hydrated.

**Figure 2:**
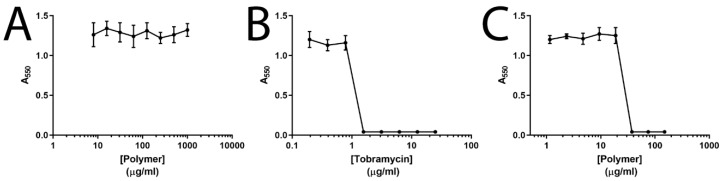
Minimum inhibitory concentration (MIC) analysis of tobramycin-loaded alginate/chitosan particles against *P. aeruginosa*. (**A**) Unloaded particle control. (**B**) Free tobramycin. (**C**) Tobramycin loaded particles. Mean values ± S.D, *N* = 3.

Tobramycin loading (Table 2) and release (Figure S3) was quantified by fluorimetry after derivatisation of the tobramycin with ortho-phthaldialdehyde [37] (the calibration curve is depicted in Figure S4); a typical biphasic release was observed with 18.9% of the entrapped tobramycin released within the first 24 h, although the overall release was limited to 25.4% of the total amount of loaded tobramycin over the course of the experiment. The optimal formulation of the tobramycin-loaded alginate/chitosan particles was tested against live cultures of *P. aeruginosa* (Figure 2). The unloaded particles showed no activity against *P. aeruginosa*, tobramycin alone had a Minimum inhibitory concentration (MIC) of 1.5 µg/mL, and the tobramycin-loaded particles showed activity in a dose dependent manner as expected, with a somewhat elevated MIC of 6.25 µg/mL, which was likely to be due to the rate of diffusion of tobramycin from the particles. While this would still be compatible with the delivery of a clinically relevant dosage (of 300 mg twice daily) to be delivered as the biopolymers are biocompatible, future studies will be directed towards improving the release profiles by tuning the crosslinking density of the particles.

### 3.2. SLPI-Conjugated Particle Preparation and Interactions with Model Biological Milieu

Disease states such as CF are characterised by increased infiltration of pro-inflammatory cytokines in the lung [25] which leads to extensive tissue damage through NE mediated activity [12]. As a natural inhibitor of NE [31] it was anticipated that the conjugation of SLPI to the particles could potentially inhibit NE mediated tissue destruction while also achieving passive targeting for the particles. The bioconjugation was achieved via carbodiimide chemistry. The properties of particles prepared with SLPI in the absence/presence of carbodiimide crosslinker are displayed in Table 3 (and Figure S5), with particles formed in the absence of carbodiimide containing 0.2 µg of SLPI, whereas those formed in the presence of carbodiimide containing 11.2 µg of SLPI, highlighting the necessity of using the carbodiimide to attach the SLPI to the particles.

**Figure 3:**
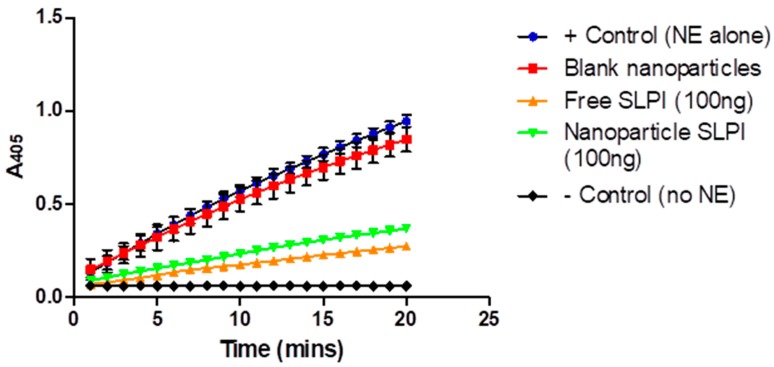
Inhibition of neutrophil elastase (NE) by secretory leukocyte protease inhibitor (SLPI)-conjugated particles. Results presented as mean ± S.D, *N* = 3.

To evaluate whether any of the conjugated SLPI was functional after conjugation to the chitosan in the particles, the SLPI-conjugated particles were incubated with human neutrophil elastase (NE) and their ability to inhibit the cleavage of a chromogenic substrate (methoxysuccinyl-Ala-Ala-Pro-Val-P-nitroanilide) was studied [38] and compared to free SLPI and unmodified particles (Figure 3). The unmodified/blank alginate/chitosan particles show very little NE inhibitory activity (similar to NE alone), by contrast the SLPI conjugated particles displayed a similar level of activity to the free SLPI, confirming that the SLPI retains NE inhibitory activity when conjugated to the particles.

**Figure 4:**
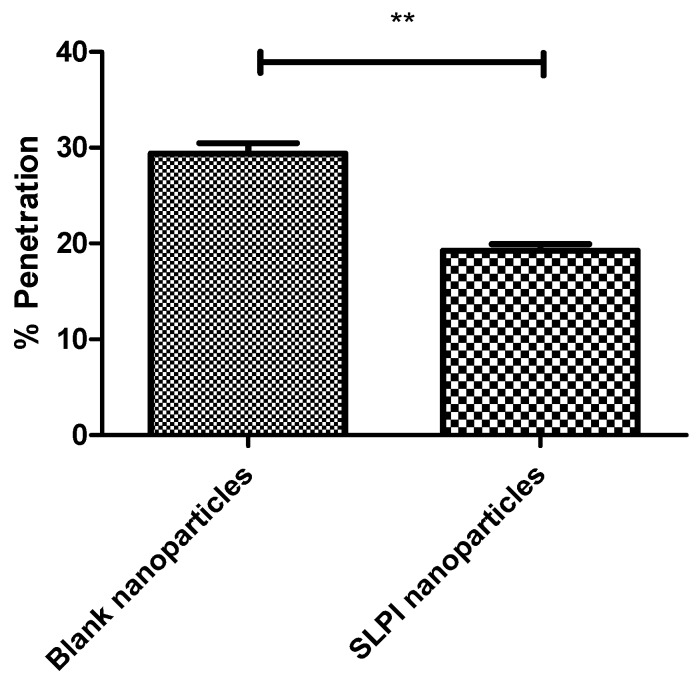
Ability of rhodamine loaded particles to penetrate CF mucus. Mean ± S.D, *N* = 3, (** *p* < 0.01).

In order to effectively treat *P. aeruginosa* infections it is necessary for drugs to achieve therapeutic concentrations at the site of bacterial colonisation. *P. aeruginosa* has been shown to reside within the thick mucus secretions within the CF lung [39]. We anticipated that the conjugation of SLPI to the particles could enhance their mucoadhesive properties via electrostatic interactions [40]. The penetration of SLPI-conjugated particles into CF mucus was assessed using rhodamine-loaded particles (Figure 4 and Figure S6) in accordance with a protocol found in the literature [41]. We observed that the SLPI conjugated particles entrapping rhodamine dye were retained to a greater level in CF sputum over the non-conjugated particles. Only 19.4% of particles functionalised with SLPI were shown to traverse CF mucus as opposed to 29.7% of particle without SLPI (in line with literature showing particles with cationic coatings could be retained within the pulmonary environment longer, thereby increasing the therapeutic efficacy of the drug) [42].

### 3.3. Applicability

Chronic infection with *P. aeruginosa* is of key importance in CF pathogenesis [13], and if treatment with colistimethate sodium is ineffective it is treated by the nebulisation of tobramycin at a dose of 300 mg twice daily [14,15,18]. Akin to many conditions, problems with CF therapy include systemic toxicity and non-compliance of the patients [19], however, its treatment is further complicated by the accumulation of thickened mucus in the pulmonary environment. Consequently, the generation of drug delivery systems capable of tailoring the therapeutic delivery paradigm has significant potential for clinical benefits [43,44,45,46,47,48,49].

Herein we have described the results of our preliminary investigation of the development of alginate/chitosan particles for tobramycin delivery to pulmonary environments. Tobramycin loading and release from the alginate/chitosan particles was investigated, with evidence of both uptake and release of sufficient tobramycin to inhibit *P. aeruginosa* in vitro; albeit with room for improvement to the quantity/speed of delivery that could be achieved by tuning the crosslinking density of the particles. Functionalisation of the alginate/chitosan particles with secretory leukocyte protease inhibitor (SLPI) was shown to help inhibit the inflammatory response associated with lung infections (via inhibition of neutrophil elastase activity) and enhance their interaction with cystic fibrosis mucus (assayed via reduction of the depth of particle penetration into the mucus) in vitro. These results highlight our capability to develop drug delivery systems which can enhance the antibacterial activity of the delivered payload through increased accumulation at the site of infection while reducing the aberrant inflammatory response to infection [11,12,23,24,25,32,33,34]. These findings have potential to be used much more broadly in the development of a variety of pharmaceutical formulations for pulmonary applications in academia and industry, with potential for eventual clinical translation after significant further research and development to optimise properties and dosing methodology.

## 4. Conclusions

The complexity of CF treatment regimens has been shown to be largely responsible for patient non-compliance [19,50], and the formulation of particles displaying mucoadhesive properties is a potential solution to minimise problems associated with non-compliance, with potentially significant beneficial economic, health and societal impacts at the global scale. The particles described herein were capable of delivering a potent antimicrobial (tobramycin) to *P. aeruginosa*, and the conjugation of SLPI enhanced their mucoadhesive properties [39,42], potentially increasing the efficacy of drug delivery over prolonged periods, and thereby potentially helping to minimise problems associated with non-compliance after successful pre-clinical and clinical studies [51,52].

## Figures and Tables

**Table 1:** Optimisation of formulation parameters in the design of alginate/chitosan particles. Results presented as mean ± standard deviation (S.D.), *N* = 3.

Alginate (*w*/*w* Ratio)	Chitosan (*w*/*w* Ratio)	Tobramycin (*w*/*w* Ratio)	CaCl_2_ (*w*/*w* Ratio)	Aggregation
9	1.5	1.5	3	Yes
9	1.5	1.5	0.8	Yes
9	0.8	0.8	0	No
9	1	3	0	Yes
9	1	1.5	0	No

**Table 2:** Properties of the particles prepared with the optimal formulation of alginate:chitosan:tobramycin (9:1:1.5, *w*/*w*/*w*). Results presented as mean ± S.D, *N* = 3.

Particle Size (nm)	PDI	Zeta Potential (mV)	Tobramycin Loading in Particles (µg/mg)	% Entrapment
437.5 ± 22.3	0.27 ± 0.07	21.6 ± 1.1	74.2 ± 3.4	44.5 ± 2.0

**Table 3:** Particle properties following preparation without/with carbodiimide. Results presented as mean ± S.D, *N* = 3.

Crosslinker (EDC)	Particle Size (nm)	Polydispersity Index (PDI)	Zeta Potential (mV)	Conjugated SLPI (µg) in Particles (mg), (µg/mg)
No	437.5 ± 26.5	0.26 ± 0.09	−22.9 ± 3.1	0.2 ± 0.3
Yes	458.0 ± 31.1	0.31 ± 0.12	−19.2 ± 2.1	11.2 ± 2.3

## Supplementary Materials

The following are available online at https://www.mdpi.com/1999-4923/11/8/379/s1, Figure S1: DLS analysis of the particles prepared with the optimal formulation of alginate:chitosan:tobramycin (9:1:1.5, *w*/*w*/*w*). Figure S2: TEM analysis of alginate/chitosan particles. Figure S3: Cumulative release of tobramycin release from alginate/chitosan particles. Figure S4: Calibration curve for tobramycin sulphate in particle supernatant following formulation of tobramycin loaded alginate/chitosan particles. Figure S5: Calibration curve for SLPI in the range (31.25–500 µg/mL) quantified with the BCA assay kit (Pierce, UK). Figure S6: Standard curve of Rhodamine 6G in the concentration range (0–1000 ng/mL).
