# Supplementary material for: Alginate/Chitosan Particle-Based Drug Delivery Systems for Pulmonary Applications
Source: Pharmaceutics. 2019 Aug 2;11(8):379. doi: 10.3390/pharmaceutics11080379 (PMC6722511; doi:10.3390/pharmaceutics11080379)
Supplement: Supplementary file 1 [file pharmaceutics-11-00379-s001.pdf]

pharmaceutics

Supplementary Materials: Alginate/Chitosan Particle-Based Drug Delivery Systems for Pulmonary Applications

Marcus Hill, Matthew Twigg, Emer A. Sheridan, John G. Hardy, J. Stuart Elborn, Clifford C. Taggart, Christopher J. Scott and Marie E. Migaud

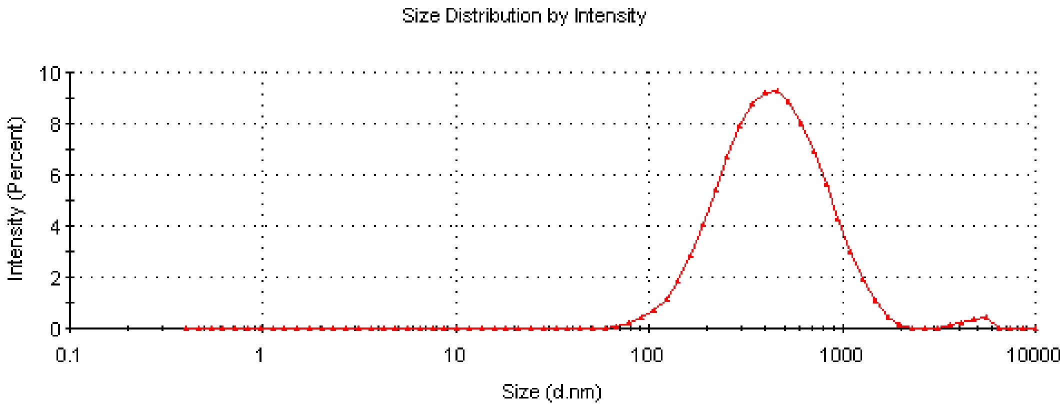

Figure S1. DLS analysis of the particles prepared with the optimal formulation of alginate:chitosan:tobramycin (9:1:1.5, *w*/*w*/*w*).

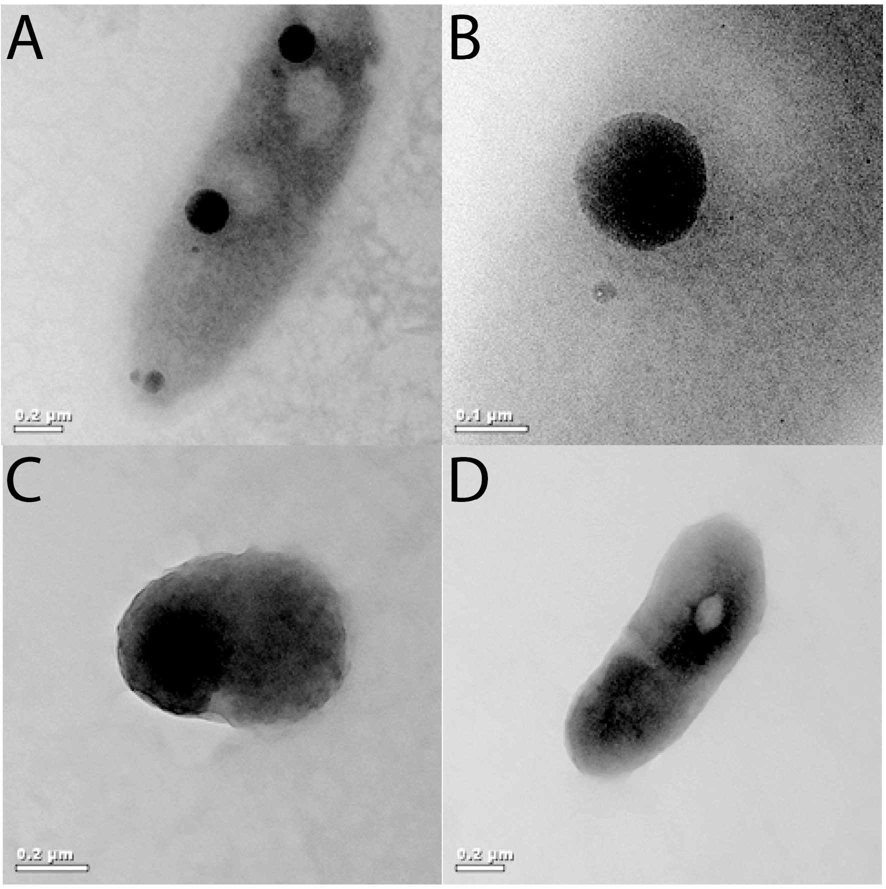

Figure S2. TEM analysis of alginate/chitosan particles. (**A**) Image showing particles observed at × 9,900 (scale bar 200 nm). (**B**) Increased resolution image at x 29,000 showing a single particle within the 100–200 nm size range (scale bar 100 nm). (**C**) and (**D**) enhanced magnification image at x 15,000 and × 9,900, respectively, showing agglomeration of individual particles (scale bar 200 nm).

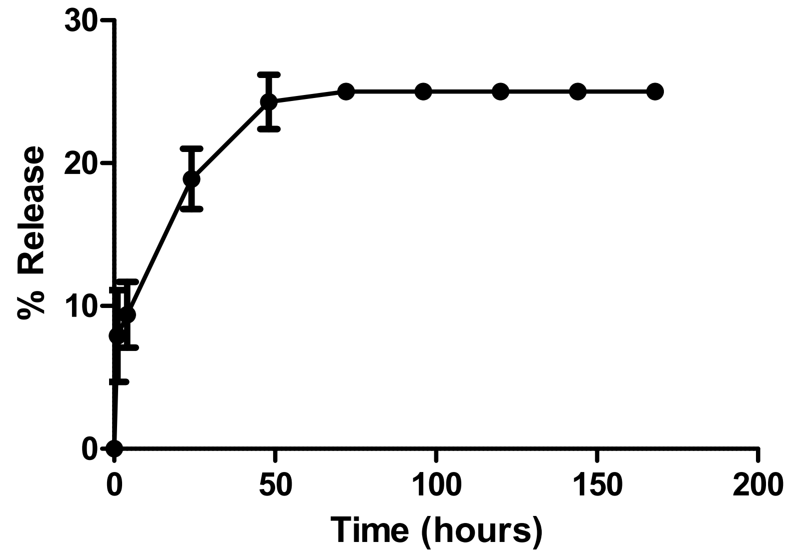

Figure S3. Cumulative release of tobramycin release from alginate/chitosan particles. Mean ± S.D, *N* = 3.

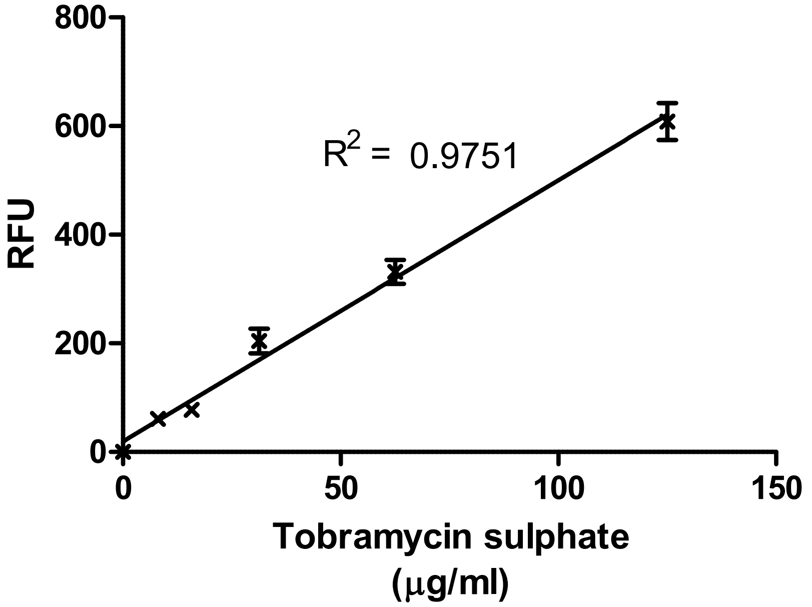

Figure S4. Calibration curve for tobramycin sulphate in particle supernatant following formulation of tobramycin loaded alginate/chitosan particles. Mean ± S.D, *N* = 3.

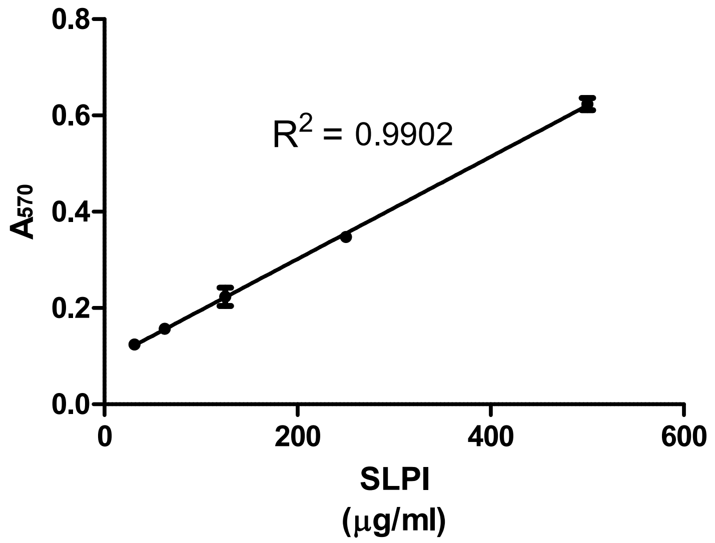

Figure S5. Calibration curve for SLPI in the range (31.25–500 μg/mL) quantified with the BCA assay kit (Pierce, UK). Mean ± S.D, *N* = 3.

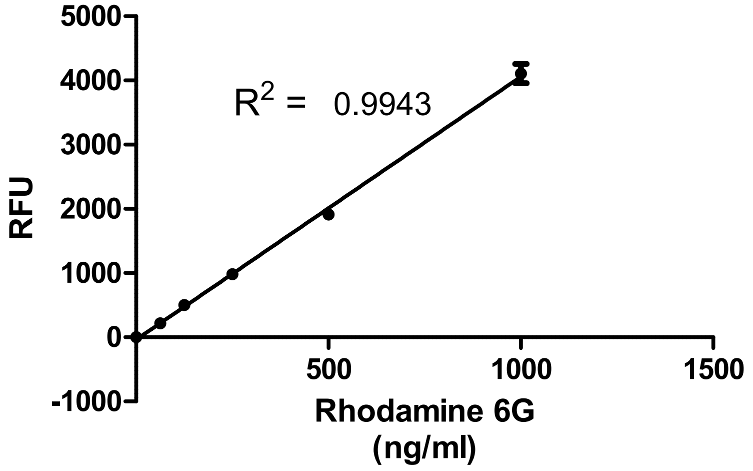

Figure S6. Standard curve of Rhodamine 6G in the concentration range (0−1000 ng/mL). Mean ± S.D, *N* = 3.
